# Primary lymphedema French National Diagnosis and Care Protocol (PNDS; Protocole National de Diagnostic et de Soins)

**DOI:** 10.1186/s13023-020-01652-w

**Published:** 2021-01-06

**Authors:** Stéphane Vignes, Juliette Albuisson, Laurence Champion, Joël Constans, Valérie Tauveron, Julie Malloizel, Isabelle Quéré, Laura Simon, Maria Arrault, Patrick Trévidic, Philippe Azria, Annabel Maruani, French National Referral Center for Primary Lymphedema

**Affiliations:** 1 Department of Lymphology and Reference Center for Rare Vascular Diseases, Cognacq-Jay Hospital, 15, rue Eugène-Millon, 75015 Paris, France; 2 Department of Genetics, HEGP, 20, rue Leblanc, 75015 Paris, France; 3 Department of Nuclear Medicine, René Huguenin-Curie Hospital, 35, rue Dailly, 92210 Saint-Cloud, France; 4 Department of Vascular Medicine, Saint-André Hospital, CHU de Bordeaux, 1, rue Jean-Burguet, 33000 Bordeaux, France; 5 Department of Dermatology and Reference Center for Rare Diseases and Vascular Malformations (MAGEC), CHRU Tours, 37044 Tours Cedex 9, France; 6 Department of Vascular Medicine, Rangueil Hospital, 1, avenue du Pr Jean-Poulhès, 31059 Toulouse, France; 7 Department of Vascular Medicine and Reference Center for Rare Vascular Diseases, CHU Montpellier, 80, avenue Augustin-Fliche, 34090 Montpellier, France; 8 Expert2expert, Paris, France; 9 Department of Internal Medicine, Saint-Joseph Hospital, 185, rue Raymond-Losserand, 75014 Paris, France; 10 Department of Dermatology and Reference Center for Rare Diseases and Vascular Malformations (MAGEC), CHRU Tours, 37044 Tours Cedex 9, France; 11 INSERM 1246 - SPHERE, Universities of Tours and Nantes, 37000 Tours, France

**Keywords:** Lymphedema, Primary, Explorations, Complications, Treatment

## Abstract

Primary lymphedema is a rare chronic pathology associated with constitutional abnormalities of the lymphatic system. The objective of this French National Diagnosis and Care Protocol (Protocole National de Diagnostic et de Soins; PNDS), based on a critical literature review and multidisciplinary expert consensus, is to provide health professionals with an explanation of the optimal management and care of patients with primary lymphedema. This PNDS, written by consultants at the French National Referral Center for Primary Lymphedema, was published in 2019 (https://has-sante.fr/upload/docs/application/pdf/2019-02/pnds_lymphoedeme_primaire_final_has.pdf). Primary lymphedema can be isolated or syndromic (whose manifestations are more complex with a group of symptoms) and mainly affects the lower limbs, or, much more rarely, upper limbs or external genitalia. Women are more frequently affected than men, preferentially young. The diagnosis is clinical, associating mild or non-pitting edema and skin thickening, as confirmed by the Stemmer’s sign (impossibility to pinch the skin on the dorsal side or the base of the second toe), which is pathognomonic of lymphedema. Limb lymphoscintigraphy is useful to confirm the diagnosis. Other causes of swelling or edema of the lower limbs must be ruled out, such as lipedema. The main acute lymphedema complication is cellulitis (erysipelas). Functional and psychological repercussions can be major, deteriorating the patient’s quality of life. Treatment aims to prevent those complications, reduce the volume with low-stretch bandages, then stabilize it over the long term by exercises and wearing a compression garment. Patient education (or parents of a child) is essential to improve observance.

## Definition

Primary lymphedema is the accumulation of lymph in the tissues, responsible for a partial or complete increase of limb volume, followed by tissue modifications, i.e., increased cutaneous thickness and fat deposition. These changes are triggered by stimulation of adipocytes and fibroblasts in response to lymph accumulation [1]. Its origin is constitutional and not the result of an iatrogenic intervention on the lymphatic system (lymph-node excision, radiotherapy), unlike secondary lymphedema. Primary lymphedema is related to hypoplasia or aplasia (rarely hyperplasia) of the lymphatic system leading to reduction of interstitial fluid absorption. It can be isolated or a component of a more complex syndrome.

This National Protocol for Diagnosis and Care (Protocole National de Diagnostic et de Soins; PNDS) addresses primary lymphedema, included in the rare vascular disease group. This protocol focuses on the diagnosis and treatment of primary lymphedema, with an additional aim of optimizing multidisciplinary management. The PNDS was coordinated by the Primary Lymphedema Referral Center of the Referral Center for Rare Vascular Diseases and developed by a group of experts including: a member of the Lymphedema and Lymphatic Malformations Referral Center, a member of the Rare and Dermatologic Genetic Disorders Referral Center (MAGEC), physicians working in highly specialized centers, two physiotherapists, two general practitioners, a nurse, a psychologist, a plastic surgeon, a podiatrist, an orthotics specialist, a member of the patient’s association and the mother of a child with primary lymphedema.

Recommendations are based on real-life experiences managing these patients in different specialized or referral centers, consensus and literature analysis. They were often extrapolated from those concerning secondary upper limb lymphedema after breast-cancer treatment. This PNDS cannot consider all specific cases: all comorbidities, hospital-care protocols, etc. It does not address the very special cases of more complex lymphatic diseases sometimes associated with lymphedema (exudative enteropathy, serous effusions). It will be updated as new data are validated. Primary lymphedema must be diagnosed by a specialist, in liaison with specialized centers and its regular follow-up coordinated with the patient’s general practitioner and other health professionals.

## Epidemiology

Primary lymphedema is a rare disease but its real prevalence is unknown in France [2]. It occurs mainly in women, with an 80% sex ratio according to old series, and ranges between 58 and 70% in the most recent series [3–5]. Primary lymphedema is more often present at birth or appears during the first year of life for boys, whereas it occurs later in girls (9–11 years), with a global female predominance [4, 6–8]. Primary lymphedema can be detected antenatally by ultrasound [9].

## Diagnosis

Primary lymphedema is often diagnosed late (> 10 years between lymphedema onset and the first clinical assessment in a specialized center), because it is confused with other diagnoses (venous insufficiency, lipedema, etc.) [10–12]. It should be suspected for a patient with unexplained distal and persistent edema. The objectives are to confirm limb lymphedema, look for potential complications and evaluate the psychological and functional impacts. A diagnosis of primary lymphedema may be advanced by a family physician, pediatrician, vascular or other medical specialist (dermatologist, cardiologist, internist, geneticist, etc.) and confirmed by a trained physician in liaison with a referral or specialized center. It should also be noted that patients can come spontaneously after consulting Referral Center websites (www.maladies-vasculaires-rares.fr, www.orpha.net), patients’ associations, hospital websites or social networks.

### Medical history

#### Adult

- Family history (primary lymphedema, chronic venous insufficiency)
- Age at lymphedema onset
- Tropical disease (travel in a country endemic for filariasis)
- Personal history of chronic venous insufficiency
- Lymphedema reversibility or not
- Past cellulitis (number of episodes, frequency)
- Social, economic, professional, psychological, esthetic impacts
- Concomitant symptoms, such as pain, potentially suggestive of another diagnosis.

#### Child

Complementary information should be sought:

- pregnancy and delivery problems: intrauterine growth retardation, prematurity
- ultrasound-detected anomalies during pregnancy: anasarca and/or serous effusions, abnormal hand and/or feet volume(s) or nuchal translucency, polymalformative syndrome.

### Initial physical examination

The following information should be collected: weight, height, body mass index (BMI) and for a child: weight/height curve, cranial perimeter. Of course, signs supporting secondary lymphedema should be sought, such as adenopathies, asthenia, weight loss, anorexia, proximal topography (thigh, pubis, genitals).

**Fig. 1:**
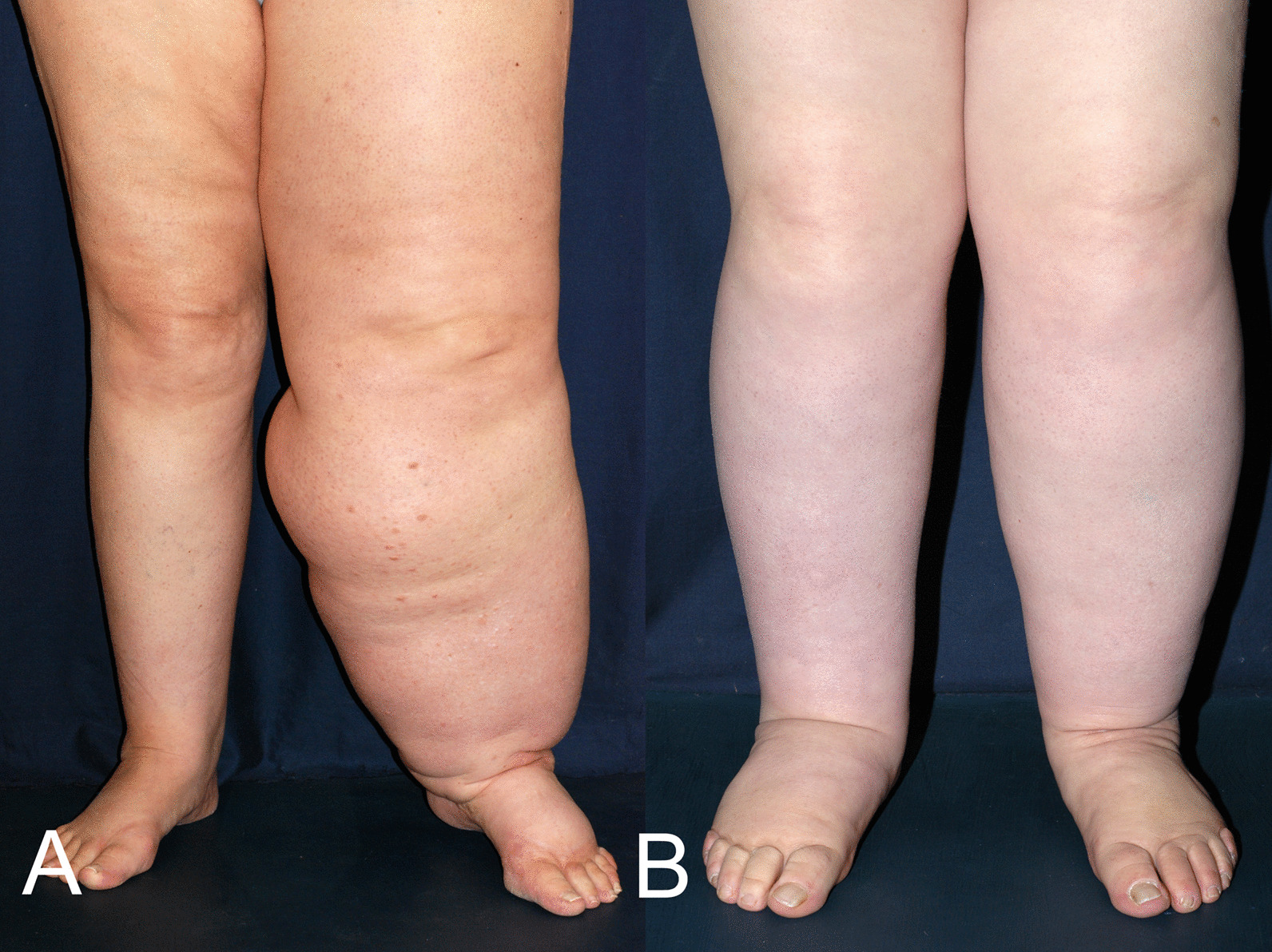
Left unilateral (**a**), bilateral (**b**) primary lymphedema

**Fig. 2:**
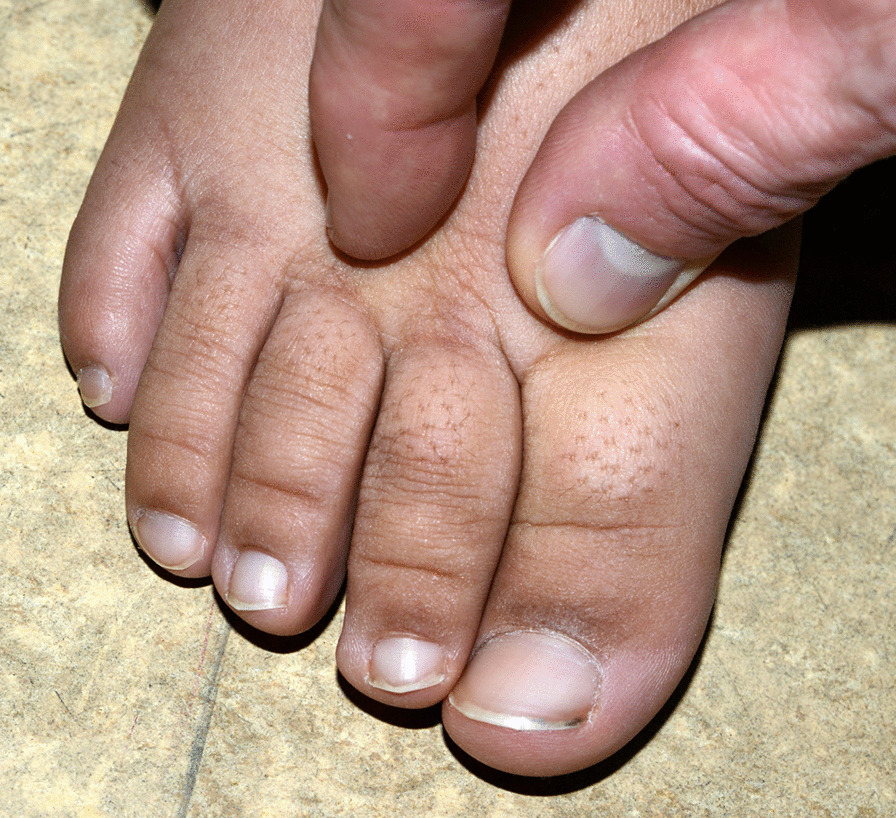
Stemmer’s sign: inability to pinch the skin on the base of the second toe

Lymphedema signs:

- Inspection: increased volume of one or more limbs (Fig. 1a, b);
- Volume (or circumference) measurements, simple (tape measure), automated, comparison of the two limbs, with constant landmarks: inferior edge of the patella, anterior fold of the elbow;
- Palpation: firm skin (thickness at pinching), with mild or no pitting edema (fibrosis);
- Stemmer’s sign (Fig. 2);
- Topography (uni- or bilateral, lower or upper limb, face, genitals);
- Genital lymphedema: man (increased scrotal skin thickness, testicular hydrocele, lymphedema of pubis, penis, foreskin), woman (lymphedema of major and/or minor labia, pubis). Lymphatic vesicles, possibly with lymph or chyle oozing.

**Fig. 3:**
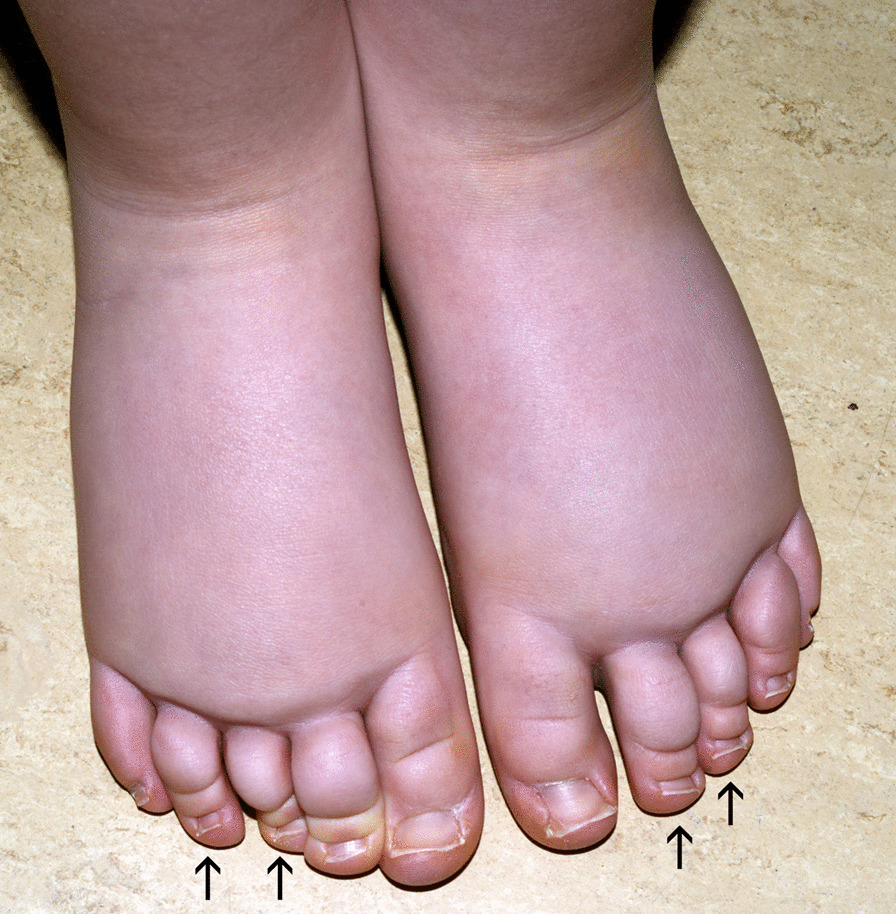
Bilateral lymphedema of a 4-year-old girl with lower-limb lymphedema present at birth, upslanting nails, brachyonychia (arrow)

Signs of lymphedema progression and complication(s):

- Cutaneous: lymphatic vesicles oozing lymph, toe papillomatosis;
- Toe-web intertrigo, ingrown nails, onychomycosis, warts;
- Nails: nail abnormalities (brachyonychia, upslanting toe nails) (Fig. 3).

Signs suggestive of a syndromic form should be sought: yellow nail(s), profuse warts, distichiasis (supernumerary row of eyelashes), vascular malformations evocative of complex vascular malformations (capillary, venous, lymphatic), hypertrophy or asymmetry of limb lengths, systemic manifestations (digestive, pulmonary, cardiac, bone), facial dysmorphia, intellectual retardation [13].

For children, specifically: clinical signs suggestive of Turner’s syndrome or another polymalformative syndrome, such as Noonan’s syndrome (particularly growth retardation for a girl and renal or cardiac malformations). Taking photos for follow-up is helpful.

## Genetic counseling

Primary lymphedema is isolated (without other associated clinical signs) or syndromic (associated with morphological, developmental abnormalities, etc.). Isolated primary lymphedema is most often sporadic [13–22]. For a patient with isolated primary lymphedema, even hereditary, the genetic cause can only be identified individually after consultation in a referral center. On the other hand, morphological or developmental abnormality found during the physical examination (facial dysmorphism, failure to thrive, growth retardation, diverse malformations) (Table 1) should lead to a specialized genetic consultation to potentially identify the underlying syndromic disease, adapt the patient’s management and offer genetic counseling to relatives. Some diseases, especially chromosomal, must be sought because their presentations may be incomplete, such as monosomy X (Turner’s syndrome), and trisomy-21 (Down’s syndrome), requiring specific care as of early childhood [23–25].

**Table 1:** Genes implicated in isolated and syndromic lymphedema forms

Syndrome	OMIM number	Associated clinical signs (non-exhaustive list)	Gene(s) implicated	Inheritance	Estimated prevalence (Orphanet 2018)
Milroy syndrome	#153100	–	*FLT4/VEGFR3*	AD	1/2500 to 1/10,000
Milroy-like syndrome	#615907	–	*VEGFC*	AD	< 1/100,000
Meige syndrome	#613480	–	*GJC2*	AD	< 1/100,000
Turner syndrome (X-monosomy)		Short stature Ovarian insufficiency Bone anomalies Deafness Cardiovascular malformations Digestive malformations Cardiac malformations	–	de novo	1/2500 to 1/10,000
Down syndrome (trisomy 21)	#190685	Facial dysmorphy Digestive malformations Skeletal malformations Cardiac malformations Extremities anomalies Hypotony	–	AD	1/2500 to 1/10,000
Noonan syndrome types 1 and 4	#163950 #610733	Arterial pulmonary stenosis Facial dysmorphy Pterygium colli (webbed neck) Learning difficulties	*PTPN11* *SOS1*	AD	1/2500 to 1/10,000
CM-AVM syndrome	#608354	Capillary malformations Arteriovenous malformations	*RASA1*	AD/mosaic	1/10,000 to 1/100,000
Lymphedema–distichiasis	#153400 #153300	Distichiasis Ungual dystrophy	*FOXC2*	AD	1/10,000 to 1/100,000
Emberger’s syndrome	#614038	Facial dysmorphy Deafness Pancytopenia Myelodysplasia	*MET* *HGF* *GATA2*	AD	1/100,000 to 1/1,000,000
Aagenaes syndrome	#214900	Neonatal cholestatic liver disease Hepatomegaly Jaundice Cirrhosis Splenomegaly Infantile malabsorption	*15q*	AR	< 1/1,000,000
Microcephaly syndrome	152950	Facial dysmorphia Microcephaly Learning difficulties Retinopathy	*KIF11*	AD (de novo)	1/100,000 to 1/1,000,000
Hennekam syndrome	#235510	Intestinal lymphangiectasia Exudative enteropathy Learning difficulties Deafness Cardiac, renal, extremity malformations	*CCBE1*	AR	< 1/1000,000
Van Maldergem’s syndrome (type 2)	# 615546	Facial dysmorphia Learning difficulties Deafness Genitourinary malformations	*FAT4*	AR	< 1/1000,000
Hereditary lymphedema type III	#616843	Facial dysmorphia Deafness Learning difficulties Lymphangiectasia	*PIEZO1*	AR	< 1/1,000,000
Oculodentodigital dysplasia	#16420	Facial dysmorphy Microcephaly Psychomotor retardation Neurological involvement Deafness Ophthalmological abnormalities (microphthalmia, cataract…) Dental anomalies Cardiac malformations Extremity anomalies	*GJA1*	AD (de novo)	< 1/1,000,000
Lymphedema-choanal atresia	#613611	Choanal atresia	*PTPN14*	AR	< 1/1,000 000
OLEDAID syndrome	#300301	Ectodermic dysplasia Osteopetrosis Immunodeficiency	*IKBKG/NEMO*	XLR	< 1/1,000,000
Hypotrichosis–lymphedema–telangiectasia syndrome	#607823 #137940	Facial dysmorphia Hypotrichosis of the scalp and face Telangiectasia Glomerulosclerosis	*SOX18*	AR/AD	< 1/1,000,000

*AD* autosomal dominant, *AR* autosomal recessive, *OLEDAID* osteopetrosis–lymphedema–ectodermal dysplasia anhidrotic with immunodeficiency, *XLR* X-linked recessive

## Complementary investigations

### Explorations

Primary lymphedema diagnosis is clinical after excluding other causes of edema. The following investigations should be obtained:

- Venous Doppler ultrasound to rule out post-thrombotic venous syndrome and look for avalvular venous insufficiency, extremely rarely associated with primary lymphedema (lymphedema–distichiasis syndrome);
- Laboratory tests: albuminemia, protein electrophoresis, proteinuria;
- Abdominal-pelvic computed-tomography (CT) scan or ultrasonography to eliminate secondary lymphedema caused by a compressive mass (increasing risk with age): when malignant disease is strongly suspected, complementary explorations may be repeated or completed with a positron-emission-tomography scan.

### Lymphatic system explorations

Lymphoscintigraphy is useful to diagnose and confirm primary lymphedema [26]. It is a low radiation examination but forbidden during pregnancy and breastfeeding. Bilateral hypodermal injections are administered between the first and second toes (or fingers in rare forms of upper limb primary lymphedema). The large size of ^99m^technetium radiolabeled nanocolloid, essentially albumin (rarely rhenium colloid) leads to it being entrapped only by lymphatic capillaries and then drained by lymphatic system. It enables comparative, functional and bilateral evaluation of the two upper or lower limbs: lymph-node uptake (groin, axillary), possible lymphostasis, possible dermal back flow, rerouting into the deep lymphatic system (popliteal or epitrochlear lymph-node visualization). Images are obtained usually after 40–45 min of exercises, sometimes later.

Other investigations that should not be prescribed systematically but may be useful for diagnosis or differential diagnosis [27]: limb CT scan or magnetic resonance imaging (MRI), hybrid-lymphoscintigraphy, non-enhanced lymph-MRI for more complex cases [28–30], high frequency skin ultrasound [31], tissue dielectric constant, bioimpedance spectroscopy (to measure extracellular water), dual-energy X-ray absorptiometry (DEXA, to measure fat and lean masses), lymphofluoroscopy with indocyanine green to visualize lymphatics in real-time, and tissue dielectric constant are, to date, still fundamental research techniques [27, 32, 33].

For a child, neither venous ultrasonography nor laboratory analyses are ordered systematically. The physical examination remains the most important determinant, with the systematic search for clinical signs of a malformative syndrome. Lymphoscintigraphy is possible in children, usually after 7/8 years of age, when they can understand their participation in the investigation. For complex malformative disease implicating the lymphatic system, MRI may be useful to assess systemic involvement and its extent.

## Lymphedema management

Lymphedema management has the primary goals of reducing then stabilizing the volume, preventing complications (cellulitis) and facilitating their management, favoring patient autonomy and improving the patient’s quality of life. Early and rapid treatment of primary lymphedema should be initiated as soon as it is diagnosed, to decrease the risk of developing irreversible tissue changes, e.g. fibrosis and fat deposition.

No oral treatment is available or recommended. Combining the bandaging techniques with meticulous skin care and patient education has proven efficacy [34–36]. Lymphedema management is classically divided in two distinct phases that Földi described in 1980s (Table 2). The first intensive phase, called complex (or complete) decongestive therapy aims to reduce lymphedema volume and teach the patient to take on self-management and preventive measures. The second phase seeks to obtain long-term stabilization of lymphedema volume. Recurrent intensive phases may be required to optimize lymphedema management. When lymphedema volume is moderate, an intensive phase is not always mandatory.

**Table 2:** Two phases of lymphedema management

Phase 1: intensive phase (volume reduction)	Phase 2: maintenance phase (volume stabilization)
Low-stretch bandages, 24 h/24 h, for 5 days to 3 weeks	Elastic compression, during the day (every day, from morning to evening)
Exercises while wearing the bandages	Low-stretch bandages overnight (3 nights/week)
Manual lymphatic drainage	Exercises while wearing the bandages
Skin care	Skin care
	Manuel lymphatic drainage, if necessary

### Health professionals implicated

After confirmation of the lymphedema diagnosis and according to each patient’s needs, management requires trained health professionals from various fields: physiotherapists, nurses, pharmacists, orthopedists, orthotics specialists, therapeutic education teams, physicians, surgeons, podiatrists, dermatologists, psychologists, dieticians, nutritionists, social workers.

Regular monitoring by professionals trained in pediatric and adult lymphedema management is mandatory. Its frequency depends on lymphedema severity, evolution and complications.

### Intensive phase: lymphedema-volume reduction

Lymphedema-volume reduction ranges from 30 to 60%, depending on the method used to measure lymphedema volume (perimetry, volumetry) [37, 38].

This phase includes:

- Low-stretch bandages: they include padding (foam, cotton, alveolus foam), overlaid with several layers of a low-stretch bandage (extensibility < 100%) [39]. Consensus international guidelines and the French Haute Autorité de Santé (HAS) [40] recommend only this type of bandages. Despite the lack of consensus in international recommendations or published comparative reports and the non-recommendation in the HAS 2011 and 2020 documents, some centers suggest adding an elastic or cohesive band over the low-stretch bandage [36, 37, 40].
- Exercises while wearing low-stretch bandages. These movements are not clearly codified but, by analogy with secondary upper limb lymphedema after breast cancer, must be progressive, aerobic, supervised by a trained coach and guided by patient’s feedback.
- Manual lymphatic drainage (MLD). This technique has not been specifically evaluated for primary lymphedema. By extrapolating from secondary upper limb lymphedema after breast cancer, the authors of the 2015 Cochrane review could not conclude as to an MLD contribution to reducing lymphedema volume but retained a small additive effect in conjunction with low-stretch bandages on moderate lymphedema volume [41].
- Meticulous care of skin and nails (moisturizer, toe-web intertrigo treatment to detect and treat potential bacterial site(s) of entry to prevent cellulitis).
- Participation in a patient-education program authorized by the Agence Régionale de Santé (ARS; Regional Health Agency) led to the acquisition of competences: theoretical understanding of lymphedema pathophysiology; technical mastery self-bandaging and how to put on the elastic garment; to initiate early treatment of cellulitis; to adapt, cope and support autonomy and observance. Educational programs require the patient’s commitment to lymphedema management, learned during the first intensive phase and applied during the maintenance phase [42].

When a child is affected, his/her parents’ involvement in the care is essential. Treatment objectives are to avoid lymphedema worsening, to prevent cellulitis, to improve quality of life and to allow life to be “the most normal possible”. Parents should make sure of proper skin care, weight control, and the child should continue to receive childhood immunizations as per the recommended schedule and be able to participate in physical activities and sports. By analogy with adults, lymphedema management is based on complete decongestive therapy [43–45]. MLD can be done by parents with a physiotherapist’s supervision once they have learned the technique. Depending on the child’s age, compressive treatment modalities must be discussed individually with the parents and child. In particular, stockings and sleeves must be changed regularly (several times a year) to accommodate the child’s growth. No consensus has been reached about the use of compression for babies or infants before toddler age. Notably, some lymphedemas can regress spontaneously. Specific therapeutic education programs have been developed for children and young adults, coordinated with care or during more prolonged training sessions with on-site stays [46].

### Maintenance phase

It includes the wearing a compression garment. A high-pressure (class 3: 20.1–36 mmHg; class 4: > 36 mmHg), custom-made, compression garment is needed to obtain the best volume stabilization. Wearing two compression garments, one on top of the other, is possible to achieve efficient high pressure [36]. Several weave types exist for compression garments: round- or flat-knitted fabric. Each has different advantages and disadvantages (thickness, rigidity, comfort, cost, etc.). In practice, flat-knit compressions are mainly used for very dysmorphic limbs or with marked cutaneous folds at the ankle or leg or when it is necessary to compress the toes individually (foot/toe covers) or the fingers (glove). Superposition of compression is possible with a round-knit garment or by combining round and flat-knit materials, but difficult to obtain with flat-knit alone.

Other components of maintenance phase include bandaging less frequently than during the intensive phase, possible use of MLD, continuous meticulous skin care and weight control [47, 48].

### Other treatments, techniques under evaluation

#### Intermittent pneumatic compression

This technique is based on the use of sequential inflatable chambers, starting distally, with a program able to adapt the inflation/deflation durations and the pressure delivered. The device designs are different, with a variable number of chambers and programs making their comparison difficult. No high-quality study has yet been published on treatment of primary lymphedema [49].

#### Other techniques, drugs

Other techniques sometimes used to treat lymphedema (K-taping, acupuncture, balneotherapy, endermology, etc.) have not been sufficiently evaluated or have not yielded results showing reduced lymphedema volume [50]. Aqua lymphatic therapy has not been evaluated. No medical treatment has proven effective at treating lymphedema. Veinotonics are not effective. Diuretics are never indicated for lymphedema and are dangerous to use for this isolated indication. Psychological management, relaxation techniques, yoga, could be useful in some situations, even though no data are available in the literature. Nocturnal and diurnal compression systems are proposed to simplify lymphedema treatment, to improve compliance and promote autonomy. These adjustable compression wraps are currently being subjected to rigorous evaluation [51, 52].

## Organization—therapeutic indications

Organization of intensive phase treatments depends on local availability. Treatment—outpatient or inpatient—lasts for 5 days to 4 weeks, depending on the patient’s personalized therapeutic objectives, lymphedema complications and locally available centers. In-hospital or outpatient treatment requires the input of a wide variety of multidisciplinary professionals (physician, physiotherapist, surgeon, nurse, pedicure/podiatrist, psychologist, therapeutic education team, dietitian, nutritionist, orthotics specialists and social workers).

All patients with primary lymphedema must have access to specialized multidisciplinary management. Most patients require intensive phase treatment followed by maintenance therapy. When the lymphedema volume is moderate, maintenance therapy alone is possible.

In the absence of dedicated studies, therapeutic approaches are not consensual and some experts prescribe treatment as soon as the diagnosis is made, others opt to adapt the indication of compression to clinical monitoring findings or even waiting until the child can walk [43]. Hospitalization is rarely necessary.

## Physical activity

By analogy with the data published on secondary lymphedema (after breast or pelvic cancer), physical activities are not contraindicated [53, 54]. They do not worsen lymphedema and do not induce infectious complications. No sport is restricted for adults or children and, moreover, its practice contributes to weight control. Pertinently, becoming overweight is to be prevented, especially for children, for whom prevention can be initiated early. Supervision by trained professionals, progressive and incremental increases of duration, repetition, intensity are required. Wearing a compression garment is recommended for adults and children, but not compulsory because the effort can be perceived as more difficult with compression.

## Complications: cellulitis (erysipelas)

Cellulitis is an acute bacterial dermo-hypodermitis caused by β-hemolytic streptococci. Lymphedema is the main risk factor for erysipelas [55, 56]. Clinical signs include: systemic signs (high fever of sudden onset, chills/rigors), local signs (redness, pain, heat, volume increase). Patients should be informed, within the framework of a therapeutic education program, of the potential risk of cellulitis so that they can adapt their behavior.

Cellulitis treatment lasts 7 days and is based on oral amoxicillin (1 g 3 times per day for adults) or pristinamycin (1 g 3 times per day) or clindamycin (600 mg 3 times a day) [57]. The parenteral route is sometimes used during the first days of treatment if severity markers (arterial hypotension, shock, etc.) are present.

The criteria for hospitalization are poor clinical tolerance, severe local signs (skin detachment, cellulitis bullosa) or the existence of other associated risk factors (diabetes, elderly or very young, other comorbidities).

For children, the treatment also relies on amoxicillin (50 mg/kg given in 3 doses for 7 days) or amoxicillin–clavulanic acid. Cellulitis occurs in children at a rate similar to that of adults with lymphedema [58]. No combination therapy (corticosteroids) is recommended; taking nonsteroidal anti-inflammatory drugs is contraindicated [57]. Fever should disappear in 48–72 h and local signs in less than 10 days; however, the limb volume can take weeks to return to its previous volume. During the acute phase, compressive treatment (low-stretch bandages, compression) should be maintained for as long as the patient can tolerate it.

### Preventive treatment

Lymphedema treatment is essential and should help prevent cellulitis recurrences. If it does recur (2–3 cellulitis episodes per year), antibiotic prophylaxis can be prescribed, in combination with treatment of the site of entry (fungal toe-web intertrigo, fissural hyperkeratosis of the heels, onychomycosis) with benzathine-benzylpenicillin (2.4 MIU every 2–3 weeks) or oral penicillin V (1 MIU, 2–3 times a day) for a prolonged duration, not yet consensually defined [57, 59]. Because the effect is only suspensive, the risk of recurrence exists after stopping antibiotic prophylaxis. Podiatric monitoring may be necessary.

## Lymphedema surgery and liposuction

Lymphedema is a chronic disease treated with physical interventions (bandages, compression). Lymphatic-reconstruction surgery has no place today in the treatment of lymphedema [60, 61]. There are three main types of surgery.

### Cutaneous resections

They have the common goal of reducing or removing lymphedematous tissue or lesions complicating lymphedema, particularly lymph vesicles or papillomatous lesions. Resection of persistent excess skin after major volume reduction facilitates the bandaging and the wearing of a compression garment, which must be pursued over the long term, as surgery is merely an additional therapeutic tool. Resection (excision-plasty) is useful for male and female genital lymphedema [62]. Sometimes, circumcision can be performed alone for discomfiting foreskin lymphedema [63].

### Liposuction

It removes subcutaneous lymphedematous tissue by aspiration, especially for secondary upper limb lymphedema. Post-operatively, the high-pressure compression garment must be worn continuously over the long term to maintain the surgical benefit. In practice, this technique has not been widely used, particularly because of the major constraint represented by the permanent wearing of such a garment [64].

### Lymphatic surgeries (reconstructive surgery)

They are intended to “repair” the damaged lymphatic system. The methodological quality of the available studies and the occurrence of potentially serious adverse events lead us to not recommend surgery as lymphedema treatment outside clinical trials.

- Lymphovenous anastomoses are the main surgical technique used on the lymphatic system worldwide [65]. In France, this technique is used very marginally.
- Autologous lymph-node transplants may come from cervical, axillary or inguinal donor sites. Few publications of rigorous methodological quality are available, with a notable lack of objective volumetric evaluation [66]. In addition, a definite risk of inducing complications exists, particularly lymphedema at the donor site, but also lymphocele, hydrocele or local hypoesthesia [67].

For children, only genital lymphedema resection can be proposed and is subject to the opinion of a referral center. Other surgical techniques have not been evaluated [68].

## Main differential diagnoses

### For adults

Other causes (cardiac, renal or hepatic diseases) should be excluded by physical examination and clinical investigations.

Lipedema is considered a clinical entity rather than a disease and is often confused with primary lymphedema. It is defined as an abnormal accumulation of adipose tissue from hips to ankles, initially leaving the foot untouched [69, 70]. Lipedema almost exclusively affects women, most often obese, and usually begins at puberty. The skin remains supple, painful when pinched or after physical contacts or shocks, even minor, and pitting edema is absent after a prolonged period (e.g., overnight or several hours). Signs of obesity-related venous insufficiency may be associated, as is spontaneous bruising.

Chronic venous insufficiency with edema could sometimes be mistaken for primary lymphedema but without Stemmer’s sign. Venous Doppler ultrasound can help make the diagnosis. Advanced forms of chronic venous insufficiency may exhibit lymphatic overload but venous insufficiency signs are at the forefront.

### For children

In newborns, it is sometimes difficult to diagnose primary lymphedema when the foot and lower leg are chubby; the diagnosis becomes clearer over the following months or years. Hamartomatous or vascular anomalies may manifest as limb hypertrophy; children should be oriented towards a referral center if lymphedema is suspected, for complementary investigations (MRI, etc.) and management [10]. For post-pubertal female adolescents, lipedema is diagnosed as for adults [5, 71].

## Follow-up

Clinical monitoring, its frequency and its organization among general practitioner, specialist and physiotherapist depend on the lymphedema-evolution profile, which varies from one patient to another. Specialized follow-up also depends on the patient’s motivation and involvement in his/her own treatment. The objectives are to stabilize lymphedema volume over the long-term, ensure the patient’s treatment compliance and autonomy in coping with lymphedema, adapt the treatment to the excess volume, occurrence of complications (cellulitis, genital involvement, etc.), ensure continuity of child/adult care and take into account the psychological impact of lymphedema.

Questioning the patient during follow-up includes: episode(s) of cellulitis (number, treatment), treatment adherence according to patient’s objectives and lifestyle: wearing of the compression garment (with regular replacement), bandages, impact on quality of life, clothing, body image, sexuality for patients with genital involvement (possible consultation with a sexologist) [72–74], psychological impact with isolation, incomprehension (use of peer groups, one-on-one discussions), aesthetic impact (consult a medical clothing designer) and functional impact: joint pain, footwear (therapeutic or orthopedic shoes).

Physical examination includes volume measurement: main criterion for adults, more difficult to grasp for children because of growth, but is nevertheless useful for unilateral lymphedema, weight, appearance of the affected limb, skin suppleness, cutaneous complications (papillomatosis, hyperkeratosis, vesicles).

## Role of patient-support groups

Patient-support groups have several roles:

- to provide patients and their relatives with information in printed documents and information reviews, by organizing information meetings, with the participation of professionals, throughout France, thus participating in their education, raising public awareness and disseminating information to non-specialist professionals on lymphedema;
- to organize therapeutic education-program workshops, day meetings/sessions or short-term stays promoting lymphedema self-management (self-massage and self-monitoring) coordinated with professional caregivers;
- to create meeting spaces and telephone hotlines, allowing them to exchange experiences;
- to contact or initiate interactions with public authorities to improve patient management and quality of life;
- to represent the patients in the various health-dedicated institutions;
- to stimulate and contribute to financing for research on lymphedema and its treatment.

## Data Availability

Data sharing not applicable to this article as no datasets were generated or analyzed during the current study.
